# Vitamin K antagonist therapy: changes in the treated populations and in management results in Italian anticoagulation clinics compared with those recorded 20 years ago

**DOI:** 10.1007/s11739-017-1678-9

**Published:** 2017-05-13

**Authors:** Gualtiero Palareti, Emilia Antonucci, Ludovica Migliaccio, Nicoletta Erba, Francesco Marongiu, Vittorio Pengo, Daniela Poli, Sophie Testa, Alberto Tosetto, Armando Tripodi, Marco Moia, On behalf of the centers participating in the FCSA-START-Register (The ISCOAT 2016 study: Italian Study on Complications of Oral Anticoagulant Therapy-2016), Sophie Testa, Oriana Paoletti, Giuliana Guazzaloca, Daniela Poli, Rossella Marcucci, Vittorio Pengo, Anna Falanga, Teresa Lerede, Antonietta Piana, Francesco Cibecchini, Giuliana Martini, Lucia Ruocco, Simona Pedrini, Lucilla Masciocco, Pasquale Saracino, Angelo Benvenuto, Claudio Vasselli, Eugenio Bucherini, Andrea Toma, Pietro Barbera, Antonio Insana, Carmelo Paparo, Serena Rupoli, Giuseppe Malcangi, Rossella Sangiorgio, Maddalena Loredana Zighetti, Catello Mangione, Enrica Agostinelli, Walter Ageno, Vincenzo Oriana, Nicola Lucio Liberato, Paola Casasco, Alberto Tosetto

**Affiliations:** 1 Cardiovascular Diseases, University of Bologna, Coordinator of the START-Register, Bologna, Italy; 2 Arianna Anticoagulazione Foundation, Via Paolo Fabbri 1/3, 40138 Bologna, Italy; 3 Dip Medicina di Laboratorio, Patologia Clinica-Merate, Asst Lecco, Lecco, Italy; 4 UO Medicina Interna ed Emocoagulopatie, Policlinico Univeristario, Cagliari, Italy; 5 Department of Cardiothoracic and Vascular Sciences, University Hospital of Padua, Padua, Italy; 6 Thrombosis Centre, Department Heart and Vessels, University Hospital of Florence, Florence, Italy; 7 Haemostasis and Thrombosis Center, Department of Laboratory Medicine, AO Istituti Ospitalieri, Cremona, Italy; 8 Department of Hematology, S. Bortolo Hospital, Vicenza, Italy; 9 Angelo Bianchi Bonomi Hemophilia and Thrombosis Center, Department of Clinical Sciences and Community Health, Università degli Studi di Milano, IRCCS Cà Granda Maggiore Hospital Foundation, Milan, Italy

**Keywords:** Atrial fibrillation, Bleeding, Thrombotic, Venous thromboembolism, Warfarin

## Abstract

Vitamin K antagonists (VKA) are the most widely used anticoagulants in the world. An appropriate management of treated patients is crucial for their efficacy and safety. The prospective, observational, multicenter, inception-cohort FCSA-START Register, a branch of START Register (NCT02219984) included VKA-treated patients managed by centers of Italian Federation of anticoagulation clinics (AC). Baseline patient characteristics and data during treatment were analyzed and compared with those of ISCOAT study, performed by the Federation and published in 1996/7. 5707 naïve patients [53% males, mean age 73.0 years (28.1% >80 years)], 61.6% treated for atrial fibrillation (AF), and 28.0% for venous thromboembolism were included. During the 8906 patient-years (pt-yrs) of observation, 123 patients had major bleeding (MB) (1.38% pt-yrs; fatal: 0.11% pt-yrs), while non-major clinically relevant bleeds were 144 (1.62% pt-yrs). Bleeding was more frequent in elderly (≥70 years; *p* = 0.04), and during initial 3-month therapy (*p* = 0.02). Bleeding rate was 2.5% pt-yrs for temporally related INR results <3.0, increasing to 12.5% for INR ≥ 4.5. Thrombotic events were 47 (0.53% pt-yrs; 4 fatal 0.04% pt-yrs). Compared with ISCOAT-1996/7 results, patients older than 80 y are increased from 8 to 28% (*p* < 0.01), and those treated for AF are increased from 17 to 61%. The quality of anticoagulation control and incidence of MB are not different. However, thrombotic complications fell drastically from 3.5 to 0.53% pt-yrs (*p* < 0.01), with lower mortality (*p* = 0.01). VKA-treated patients monitored in Italian AC have good clinical results, with low bleeding and thrombotic complications rates. Important changes in the treated population and improvement in thrombotic complications are detected compared with the ISCOAT-1996/7 study.

## Introduction

In 1996 the ISCOAT study was published in The Lancet [1] focused on management and bleeding complications associated with chronic anticoagulation with vitamin K antagonists (VKA); the results regarding thrombotic complications were published in the subsequent year [2]. To our knowledge, the ISCOAT study was at that time one of the first studies on patients treated with VKA anticoagulation designed and performed according to rigorous methodological criteria. It was a large, prospective, observational, multicenter, inception cohort study on the real-life management of VKA-treated patients who were monitored by means of the INR system (established in 1985 [3]) in dedicated anticoagulation clinics belonging to the Italian Federation of Anticoagulation Clinics (FCSA). After 20 years, VKA are still the most widely used anticoagulant drugs in the world. It is fair to surmise, however, that many aspects relating to their use and clinical results may now be changed.

In the present study, we aim at assessing the characteristics of naïve patients who started VKA-anticoagulation, and were monitored in FCSA centers participating in the FCSA-START-Register, and clinical results achieved during follow-up. We also compare the currently observed results with those of the ISCOAT study published 20 years ago.

## Methods

### The FCSA-START register

The present study is based on data collected in the FCSA-START Register, which is a branch of the START Register (NCT02219984) open to anticoagulation clinics affiliated with the FCSA. The START-Register is an observational, prospective, multicenter, dynamic cohort study that includes adults (≥18 years) who start anticoagulation therapy, whatever the drug and dosage used, and who are naïve to previous anticoagulant treatment. The aim of the registry is to collect data on the incidence of adverse events in patients taking anticoagulants, the impact on quality of life and patient compliance with treatment. The characteristics of the START-Register and the rules for participation are detailed elsewhere [4].

### Patients, data collection and study monitoring

Recruitment of patients in the FCSA-START Register began in January 2012 and is still ongoing. Only patients aged 18 years or more receiving for the first time and for no more than 30 days either warfarin (Coumadin-R) or acenocoumarol (Sintrom-R), the two VKAs commercially available in Italy, are included in the present analysis. For the purposes of this study, analysis of each patient’s observation period started on the day of inclusion and ended on 31 December 2015, or sooner if a major bleeding event occurred, if treatment was discontinued for any reason or if the patient stopped attending the center.

The collected baseline data include demographic and clinical characteristics of patients, associated risk factors for bleeding and thrombotic complications, routine laboratory data, clinical indication for treatment, therapeutic range expected, and use of concomitant drugs. Serum creatinine levels are measured by local hospital laboratories, and creatinine clearance (CrCl) is calculated by the Cockcroft-Gault formula [5]. Renal failure is defined according to National Kidney Foundation stratification [6]. Patients with non-valvular AF are stratified for stroke risk evaluation according to CHADS2 [7] and CHA2DS2VASc [8] scores, while baseline bleeding risk is evaluated using HAS-BLED score [9]. In patients with venous thromboembolism (VTE), the assessment of type, site, and presence of risk factors is mandatory; the presence of biochemical or molecular risk factors is optional.

The follow-up data include all information regarding the management of VKA treatment, and events or complications occurring during treatment. As regards the quality of anticoagulation laboratory control, the time spent within, below, or above the INR range 2–3 (Time in Therapeutic Range, TTR), is computed according to the Rosendaal’s method [10], after exclusion of the first 3 months of therapy. The same program was used in this study to calculate the incidence of events in different categories of achieved intensities of anticoagulation by dividing the number of events occurring in patients with temporally related INR values in each category by the total number of patient-years accumulated in that category. Using the same criteria adopted in the ISCOAT 1996/7, an INR value is considered as “temporally related” to a bleeding or to a thrombotic event when it is obtained at the time of the event, or during the preceding 8 or 15 days, respectively.

Clinical outcomes are major bleeding complications, defined as recommended by the ISTH [11], and thromboembolic events, defined as clinically verified stroke/thromboembolism/TIA, VTE (DVT and or PE), or superficial vein thrombosis (SVT) or myocardial infarction. Non-major but clinically relevant (NMCR) bleeding events are also recorded, defined in accordance with the recent recommendation by the SSC of the ISTH [12]: bleeds that do not satisfy the criteria for major bleeding but require medical intervention by a healthcare professional, or lead to hospitalization or increased level of care, or prompt a face to face evaluation, or lead to dosage changes of the anticoagulant drug. In the FCSA-START Register all the patient’s clinical features and data regarding VKA management are recorded on web-based CRF (case report forms), and all information is electronically stored in an anonymous form in the central database of the Register. The completeness of baseline and follow-up data is checked remotely by the central monitor of the Register.

### Statistical analysis

Descriptive analysis is performed. Continuous variables are expressed as median and interquartile range (IQR) or as mean ± standard deviation (SD). Categorical variables are expressed as frequencies and percentages. Differences between groups are assessed by the Fisher’s exact text Chi-square test. The incidence of major and NMCR bleeding events was calculated, separately and all together. Data were censored after the first major bleeding complication, after the cessation of OAT or when a patient stopped being monitored by the FCSA center.

The independent effect of various possible risk factors (gender, age, indication for anticoagulation, occurrence of bleeding events from the beginning of treatment and achieved anticoagulation intensity) was investigated by performing a Poisson regression analysis. The SPSS software for Windows, version 19 (SPSS Inc, Chicago, IL, USA) is used for data processing.

## Results

### Study population

A total of 5707 VKA-treated patients (53% males) were included in the registry by 27 anticoagulation clinics. The demography of the patients, their characteristics and follow-up are shown in Table 1. At inclusion, the mean age of patients was 73.0 years, with 28.1% >80 years. The most frequent indication for anticoagulation was atrial fibrillation (61.6%; AF), followed by VTE (28.0%). Patients included for VTE had an index event that was idiopathic in 75% of cases; among the remaining, active cancer was the most frequent clinical condition (7.5%), followed by surgery (5.8%), prolonged (> 4 days) bed resting (3.4%), immobilization (within 3 months; 2.4%) and other conditions. The total follow-up period was 8906 years, whereas the individual median follow-up was 16 months (IQR 7–26). During the observation period, 351 (6.1%) patients died for reasons other than anticoagulation, with no real differences for gender and treatment indication. Anticoagulant treatment was stopped in 1758 (30.8%) patients, with 780 treated for AF and 710 for VTE. Sixty-six patients (1.2%) were lost to follow-up.

**Table 1:** Demography, patient characteristics and follow-up

	*N* (%)	Person-years of follow-up
All patients	5707	8906
Males	3029 (53)	4744
Age, mean (± SD) years	73.0 (19.0)	
Age* n* (%) < 70	2069 (36.2)	2930
≥70	3638 (63.8)	6321
>80	1605 (28.1)	2585
Indication for anticoagulation		
Atrial fibrillation	3516 (61.6)	5907
Venous thromboembolism	1593 (28.0)	2223
Heart-valve prosthesis	219 (3.8)	229
Other	379 (6.6)	150
Medical history		
No comorbidity	1071 (18.8)	
Hypertension	3945 (69.1)	
Coronary artery disease	927 (16.2)	
Diabetes	893 (15.6)	
Previous stroke/TIA	674 (14.8)	
Heart failure	654 (11.5)	
Other	1478 (25.8)	
Renal function (CrCl)		
>60 ml/min	3436 (60.2)	
30–60	1940 (34.0)	
<30	331 (5.8)	
Co-medications		
None	1360 (23.8)	
Number of associated drugs (*n*)		
1–3	2262 (39.6)	
4–5	1334 (23.4)	
>5	751 (13.2)	
Patients who stopped anticoagulant treatment	1758 (30.8)	
Died	351 (6.1)	
Lost to follow-up	66 (1.2)	
Quality of anticoagulation control		
Median (IQR) percent time spent in relation to the therapeutic range (2.0–3.0 INR)		
Below	21.0 (12.0–33.0)	
Within (TTR)	66.0 (53.0–77.0)	
Above	9.0 (3.0–16.0)	

*AF* atrial fibrillation, *FU* follow-up, *IQR* interquartile range, *TTR* percentage of time spent within the therapeutic range, *VTE* venous thromboembolism, *SD* standard deviation

### Anticoagulation control

Almost all patients were treated with warfarin (Coumadin^®^) as AVK drug, and only 124 (2.2%) patients received acenocoumarol (Sintrom^®^). The average time between two INR measurements was 19 days (Standard deviation-SD 11.3 days). The intended therapeutic range was 2.0–3.0 INR in almost all patients since only 71 patients with mechanical heart valve prosthesis had 2.5–3.5 INR. The median TTR value was 66% (IQR 53–77%), whereas 21% (12–33%) of time was spent below and 9% (3–16%) above the 2.0–3.0 INR therapeutic range.

### Bleeding complications

During follow-up, major bleeding (MB) events occurred in 123 patients (1.38% annually), 10 of them being fatal (0.11% annually) (Table 2). The hemorrhages were intracranial in 38 cases (7 fatal), digestive in 29 (3 fatal). The incidence of bleeding was not statistically different in males and females (1.48 and 1.24% annually, respectively). It was significantly higher in patients aged 70 years or older than in those <70 years [1.55 vs 1.0% annually, respectively; relative risk (RR) 1.50 (95% CI 1.0–2.37; *p* = 0.04)], and during the first 3 months of treatment [2.1 vs 1.26% annually, respectively; RR 1.68 (1.1–2.6; *p* = 0.02)]. Though the difference was not statistically significant, the incidence of major hemorrhages increased from 1.0% annually in VTE patients, to 1.4% annually in AF patients and to 1.8% annually in those with other indications. A total of 144 (1.62% annually) non-major but clinically relevant bleeding (NMCRB) events occurred during follow-up (listed in Table 2).

**Table 2:** Bleeding and thrombotic complications during follow-up

Events* n* (rate % annually, CI)	Bleeding complications	Thrombotic complications
Major events	123 (1.38; 1.1–1.6)	47 (0.53;0.4–0.7)
Fatal	10 (0.11;0.06–1.2)	4 (0.04; 0.02–0.1)
	Intracranial 38 (0.43; 7 fatal)	Stroke 12 (0.13; 4 fatal)
	Digestive 29 (0.33; 3 fatal)	TIA 12
	Hematuria 7 (0.08)	AMI 9 (0.10)
	Hemarthrosis 3 (0.03)	Recurrent VTE 7
	Other 45 (0.50)	SVT 5
		Arterial embolism 2
Sex		
Males	71 (1.48; 1.1–1.8)	22 (0.46; 0.3–0.7)
Females	52 (1.24;0.09–1.6)	25 (0.60;0.09–1.6)
Age		
<70	30 (1.0; 0.7–1.4)	17 (0.58; 0.4–0.9)
≥70	93 (1.55; 1.2–1.8)	30 (0.50; 0.3–0.7)
RR	1.50 (1.0–2.4) *p* = 0.04	
Indication		
VTE	23 (1.0; 0.7–1.5)	15 (0.67; 0.4–1.0)
AF	86 (1.4; 1.1–1.8)	27 (0.46; 0.3–0.7)
All Others	14 (1.8; 1.0–3.0)	5 (0.64;0.3–1.5)
Timing of events (days)		
≤90* n* (% annually; CI)	28 (2.1; 1.5–3.2)	10 (0.8)
>90	95 (1.26; 1.0–1.5)	37 (0.48)
RR	1.68 (1.1*–*2.6) *p* = 0.02	
Non-major clinically relevant bleeding events *n* (% annually)	144 (1.62; 1.4–1.9)	
	Haematoma 40	
	Haematuria 27	
	Nosebleed 23	
	Anal bleeds 18	
	Metrorrhagia 10	
	Gastrointestinal 7	
	Other 19	

**Fig. 1:**
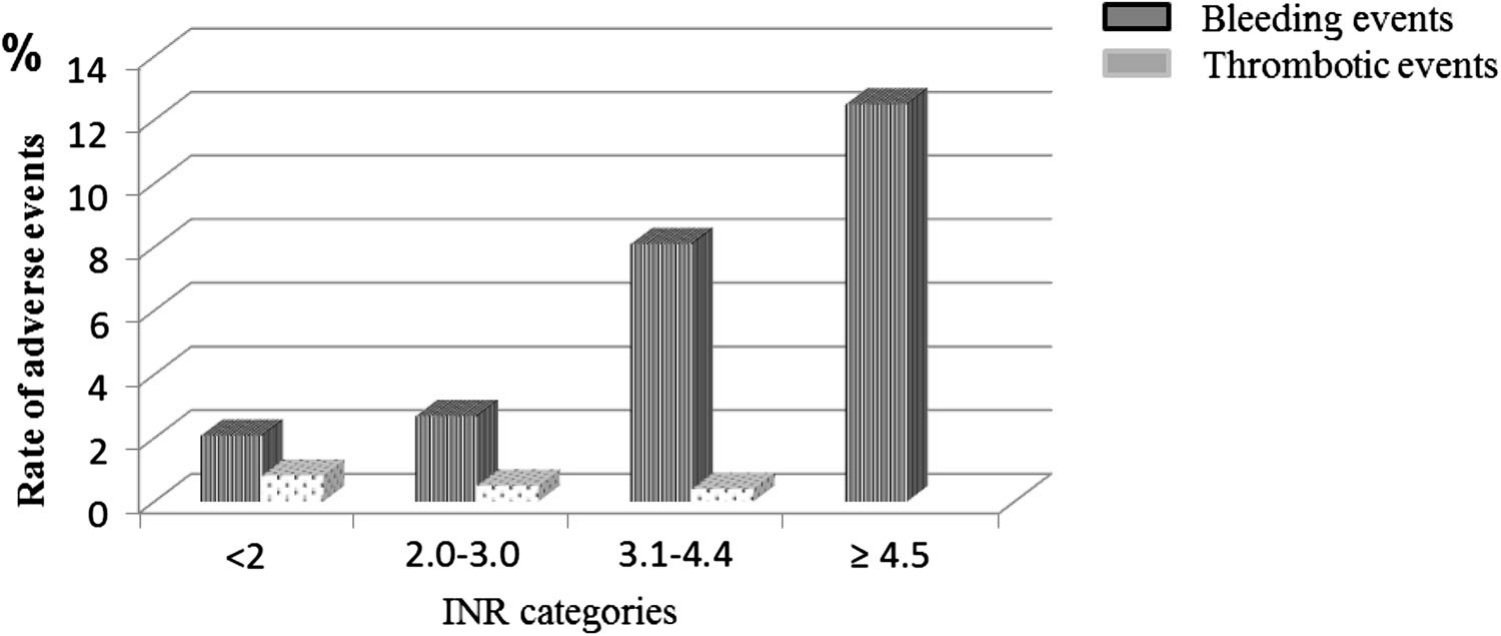
Rates of bleeding and thrombotic events in relation to the total time spent in categories of increasing INR levels

The frequency of bleeding complications in relation to anticoagulation intensity was investigated by examining the number of events in patients with temporally related INR occurring in different categories of increasing INR values; the number of events in these categories was then divided by the total number of patient years with temporally related INR results accumulated in each category. The rate of MB + NMCRB was <3% annually for INR categories <3; increasing to 6.7% annually for INR levels between 3.0 and 4.4, and to 12.5% for INR ≥ 4.5. The relative risk of INR values >3.0 vs ≤3.0 was 3.68 (95% CI 2.66–5.01; *p* < 0.0001) (Table 3; Fig. 1).

**Table 3:** Frequency of bleeding (major + non-major clinical relevant) and thrombotic (major + minor) events in categories of increasing INR levels according to the available temporally related INR results

INR categories	Patient-years of follow up with temporally related INR results	Events with temporally related INR results *n* (% annually; CI)
Bleeding events		
		*n*. 267 (32 NA)
<2.0	2157	45 (2.1; 1.5–2.7)
2.0–3.0	4960	135 (2.7; 2.2–3.2)
3.1–4.4	599	49 (8.1; 6.2–10)
≥4.5	48	6 (12.5; 5.8–24.7)
RR > 3.0 vs ≤3.0 INR (95% CI)		3.68 (2.66–5.01) *p* < 0.0001
RR ≥ 4.5 vs <4.5 INR (95% CI)		4.23 (1.53–9.75) *p* < 0.01
Thrombotic events		
		*n*. 47 (5 NA)
<1.5	340	4 (1.18;0.5–2.9)
1.5–1.99	990	7 (0.70; 0.3–1.4)
2.0–3.0	4960	26 (0.52; 0.3–0.7))
>3	1093	5 (0.45; 0.2–1.1)
RR < 2.0 vs ≥2.0 (95% CI)		1.61 (0.73–3.30) *p* < 0.01

*RR* relative risk, *CI* 95% confidence interval, *NA* not available

### Thrombotic complications

Forty-seven (0.53% annually) thrombotic complications (Table 2) occurred during follow-up, fatal in four cases (0.04% annually) with AF. The events were not differently distributed in relation to gender, age, indication for anticoagulation and timing of occurrence from the start of treatment. The rate of thrombotic complications for temporally related INR values <1.5 was 1.47% annually, decreasing in higher INR categories; the relative risk for INR values <2 vs ≥2 was 1.92 (0.92–3.78; *p* = 0.06) (Table 3; Fig. 1).

### Comparison between the current study and the ISCOAT 1996/7 results

**Fig. 2:**
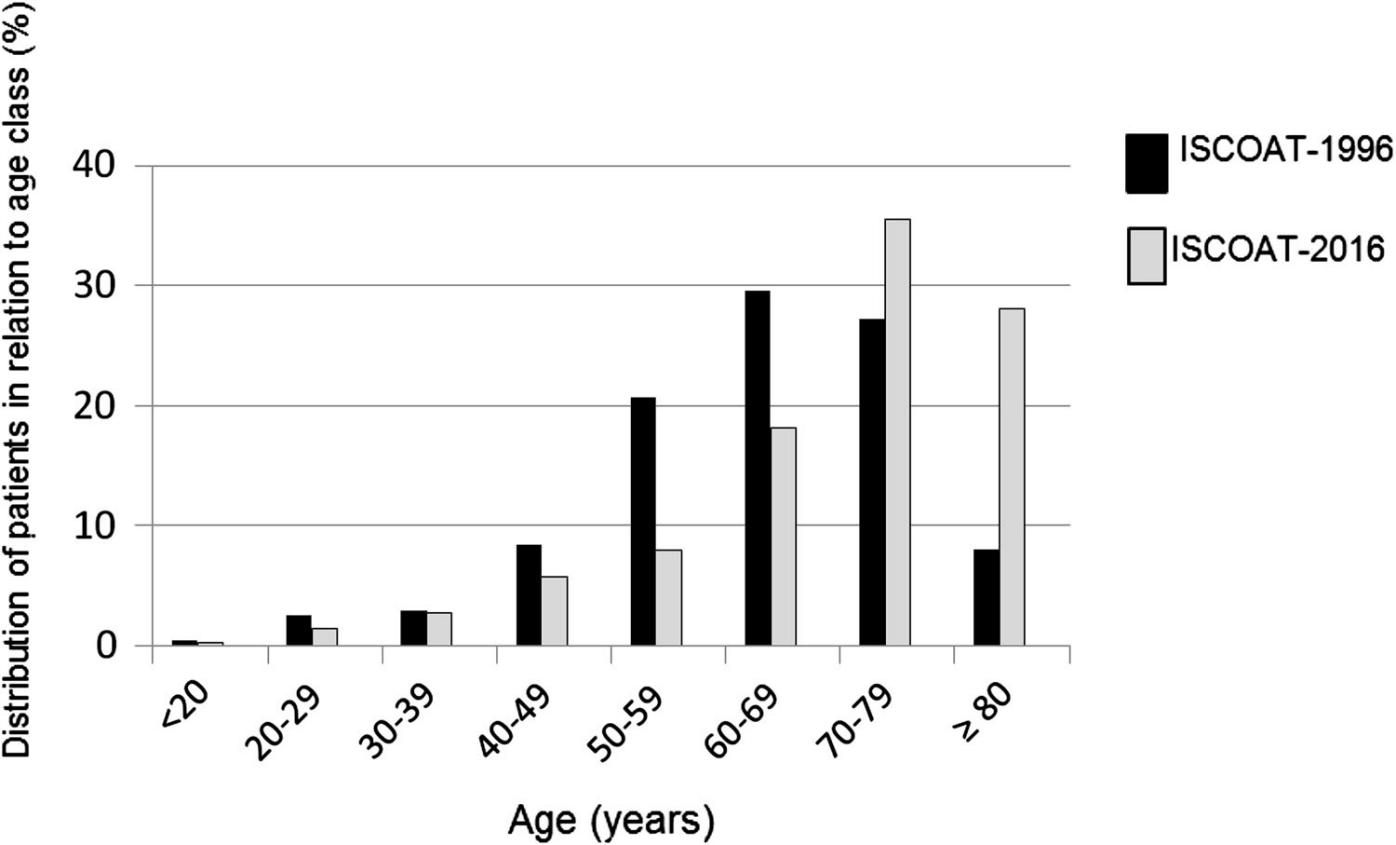
Distribution of patients in the current study and in the ISCOAT 1996/7 in relation to age

In the current study (Table 4) the patients are significantly older than those who started VKA anticoagulation 20 years ago, with an average difference of more than 10 years. The proportion of those aged >80 years increased from 8 to 28% (*p* < 0.01) (Fig. 2). This sharp increase is substantially due to the increase in patients treated for AF who represent more than 61% of all included patients, whereas they accounted for only 17% in the 1996 report (*p* < 0.01). VTE, that scored as first indication in 1996, is now in second place. Patients with arterial indications, including ischemic heart disease, who numbered about one-fourth of all patients in 1996 are now but a few, included within the “other” indications. The proportion of patients treated for heart-valve prosthesis or disease are currently less than half those of 20 years ago (6.2 vs. 17.5%; *p* < 0.001). The quality of anticoagulation control is quite similar between the two studies, with a median TTR of 66.0% versus that of 68.0% in 1996.

**Table 4:** Relevant similarities or differences between the current results (ISCOAT 2016) and those of the ISCOAT study published in 1996/7

	ISCOAT 2016, patients* n* 5707	ISCOAT 1996/7, patients* n* 2745	*p*
Age, mean (SD) years	73.0 (19.0)	63.6 (8.9)	0.01
Age			
<70	36.2	64.8	0.01
≥70	63.7	35.2	0.01
>80	28.1	8.0	0.01
Primary indication for anticoagulation %			
Venous Thromboembolism	28.0	32.5	0.01
Atrial fibrillation	61.6	16.8	0.01
Ischaemic heart disease	NA	14.7	
Arterial vascular disease	NA	10.2	
Heart-valve prosthesis/disease	6.2	17.5	0.01
Other	4.2	8.3	0.01
Quality of anticoagulation control			
Median percent time spent in relation to the intended therapeutic range:			
Below	21.0	26.1	
Within (TTR)	66.0	68.0	
Above	9.0	5.9	
Major bleeding *n*. (% annually) [fatal]	123 (1.38)	28 (1.39)	
Fatal	10 (0.11)	5 (0.25)	
ICH	38 (0.43) [7]	9 (0.45) [5]	
Gastrointestinal	29 (0.33) [3]	7 (0.35) [/]	
Other	56 (0.63) [/]	12 (0.60) [/]	
Major + NMCRB events occurring during the first 90 days of treatment *n*/*N* (%)*	78/267 (29.2)	62/153 (40.5)	0.02
Thrombotic events *n*. (% annually)	47 (0.53)	70 (3.5)	0.01 RR = 6.5 (CI 4.5–9.7) <0.01
Fatal *n* (% annually)	4 (0.04)	20 (1.0)	0.01
In pts with VTE indication	17 (0.8)	27 (4.8)	0.01
Events occurring during the first 90 days of therapy* n*/*N* (%)	10/47 (21.3)	36/70 (51.4)	0.01
Died during follow-up *n* (%)	351 (6.1%)	102 (3.7%)	0.01

*NA* non available, *TTR* percent of time spent within the therapeutic range, *NMCRB* non-major clinically relevant bleeding

* In the ISCOAT 1996/7 bleeding events were categorized as fatal, major and minor; the number 153 includes all these bleeds

The incidence of major bleeding during follow-up is similar in both studies, with equal rates of intracranial and gastrointestinal (GI) events. The rate of fatal cases is, however, lower—though not statistically significant—than that in the previous study (0.11 vs. 0.25% annually, respectively). The rate of thrombotic complications is much lower in the present study than that reported in 1997 (0.53 vs. 3.5% annually; *p* < 0.01), as also is the rate of fatal cases (*p* = 0.01). Thrombotic events are significantly less frequent during the first 90 days of treatment in the current study (involving 21.3% of all the events) than in the previous one (51.4%; *p* = 0.01). Though the number of deaths due to bleeding or thrombotic complications is significantly lower in the present study versus 1996 (14 vs. 25; respectively; *p* < 0.01), significantly more patients died during this study for different reasons: 6.1 vs. 3.7%, *p* < 0.01.

The multivariate analysis of risk factors for bleeding and thrombotic events was performed in the current cohort and compared with that of ISCOAT 1996/7 (Table 5). Female gender is significantly associated with fewer events than in men (a difference not present in the ISCOAT 1996/7), whereas the higher risk for bleeding in elderly patients (≥70 years) and for either bleeding or thrombosis during the first 90 days of treatment is confirmed in both studies.

**Table 5:** Multivariate analysis of risk factors for bleeding and thrombotic events in ISCOAT 2016 compared with that in ISCOAT 1996/7

	ISCOAT 2016	ISCOAT 1996/7
Bleeding events		
Sex (females vs men)	0.57 (0.32–1.0; *p* = 0.05)	1.21 (0.86–1.70)
Age (≥70 vs <70 years)	2.01 (1.08–3.73; *p* = 0.03)	1.69 (1.21–2.37; *p* < 0.01)
Indication (arterial disease vs others)	NA	1.72(1.17–2.54; *p* < 0.01)
Actual INR (≥4.5 vs <4.5)	1.27 (0.18–8.63; *p* = 0.2)	5.96 (3.68–9.67; *p* < 0.01)
Timing of events (≤90 vs >90 days)	11.85 (3.83–36.65; *p* = 0.01)	2.5 (1.4–3.3; *p* < 0.01)
Thrombotic events		
Sex (females vs men)	0.6 (0.3–1.1; *p* = 0.11)	0.71 (0.43–1.17; *p* = 0.18)
Age (≥70 vs <70 years)	0.56 (0.29–1.1; *p* = 0.60)	1.62 (1.0–2.61) *p* = 0.04
Indication (arterial disease vs others)	NA	1.84 (1.01–3.36; *p* = 0.04)
Actual INR (<2.0)	0.9 (0.42–1.73; *p* = 0.86)	1.88 (1.16–3.07; *p* = 0.01)
Timing of events (≤90 vs >90 days)	2.1 (1.05–4.2; *p* = 0.04)	20.6 (12.7–33.5; *p* < 0.01)

## Discussion

The present study shows that patients treated with VKA in Italian anticoagulation clinics present very good clinical results, as demonstrated by the particularly low rates of complications, both hemorrhagic and thrombotic. It also shows that important changes have occurred over the last 20 years in patients treated with VKA, and in their management in our country. This is especially the case as regards the composition of the treated populations, the indications for anticoagulation, and in a marked reduction of thrombotic complications during treatment.

The global rate of major bleeding of 1.38% per year recorded in the present study compares well with the rates of 1.40–3.40% per year reported in a recent meta-analysis of randomized clinical trials in patients with AF [13], and the 1.2–2.2% per year observed in trials on direct oral anticoagulants versus standard therapy (LMWH + warfarin) in patients with VTE [14, 15]. Higher rates of major bleeding have been reported in recent population studies: 3.8% per year in a Canadian population-based cohort study [16], and of 2.24% per year in a report from the Swedish register Auricula [17]. There is a relatively low rate of major GI bleeding in our cohort, especially in comparison with that of intracranial events. Some potential explanations may be advocated for these results. First, differently than for intracranial bleeding that is always categorized as a major event, the classification of GI bleeding is not always the same in all the reports; in our study we define them as major only in relation to the blood losses or transfused units. This is why some GI bleeds are reported as NMCRB, and some minor GI bleeds (such as proctorrhagia) were not considered even as NMCRB. Second, the very high prevalence of elderly people with AF in our cohort is likely the reason for the relatively high rate of intracranial hemorrhages; whereas, the large use of drugs for gastric protection in very elderly patients treated with VKAs may be a reason for the relatively low rate of GI bleeding. The rate of NMCRB recorded in our cohort is rather low (1.62% annually). It cannot be excluded that this low rate is, at least in part, due to underreporting. It should be pointed out, however, that the completeness of the imputed data was constantly controlled by the monitor of the registry, who also read all the information added to the electronic database by the treating doctors. It is difficult to compare our result with others in the literature since in some cases this information is not reported, or the criteria adopted for classifications are different. When reported directly, or derivable from the data shown, the rates of NMCRB in the warfarin treated patients included in phase III trials on direct oral anticoagulants in AF or VTE patients vary largely: from 2.2% [18] to 11.4% [19].

As regards thrombotic complications, the rate of 0.53% per year (0.67 and 0.46% in VTE and AF patients, respectively) recorded in our study is particularly low when compared with 1.66% per year calculated in a meta-analysis of trials on patients with AF [20], or with 2.2% per year reported in trials on VTE-patients [21], or with that of 2.65% per year found in the Auricula registry [17]. The lower rates of both bleeding and thrombotic complications recorded in our study in comparison with the Auricula’s results are surprising and not easy to explain, especially because both studies include patients managed in anticoagulation clinics, with similar classification of clinical outcomes. What is more, the mean Auricula TTR is higher (76.5%) than that in our study (66.0%), a rate which is, however, within the range of 65–70% recommended for ensuring high quality of anticoagulant therapy [22]. TTR is considered a valuable tool to assess the quality of anticoagulation control which, in turn, is important to determine clinical outcomes of treated patients [23, 24]. However, recent studies fail to find a correlation between TTR and complications (at least thromboembolic) [25]. TTR results are influenced by many factors, such as geography [24], study settings (community practice, anticoagulation clinics or clinical trials) [26], patient characteristics [27], presence of co-morbidities [28], and time-period since start of treatment [29, 30]. In a large North-American retrospective cohort study on patients treated for AF the mean TTR is 53.7% overall, but it improved over time, increasing from 47.6% for patients with <6 months of therapy to 57.5% for those with ≥6 months of testing (*p* < 0.01) [30]. Since, unlike the Auricula registry, our study includes only patients who started VKA-treatment and who, therefore, had shorter treatment times, this factor may well be one potential reason for the lower TTR rate recorded in our study.

We compared the results of the current study with those obtained in the original ISCOAT study [1, 2] (Table 4). The two studies had the same design and the same intended patient population that started VKA treatment and was managed by anticoagulation clinics affiliated with FCSA. While some results remain substantially similar between the two studies, there are big differences in other respects. The treated population in the present study is significantly older (on average 10 years more, *p* < 0.01), with the proportion of patients aged >80 years up from 8% to more than 28%. This important change is associated with the sharp increase in patients treated for AF (<17% 20 years ago and now >61%). Whereas other indications, such as VTE and heart valve prosthesis or disease, are currently significantly less frequent, the presence of ischemic heart and arterial disease, that prompted treatment in about one-fourth of patients in the original 1996 study, has now almost entirely disappeared. The high prevalence of patients treated with VKAs for arterial diseases may well be the explanation for the very high rate of thrombotic complications recorded in the ISCOAT 1996/7 and not observed in the current cohort. There is now general awareness of the fact that VKAs therapy is not the best indication, and is less effective for treatment of arterial diseases. The quality of anticoagulation control is similar in both studies, with similar TTR values. However, in spite of the sharp increase in the number of very elderly patients, a population generally expected to carry greater risk of bleeding during VKA (especially intracranial), the rates of major bleedings are almost identical (an annual 1.38% now versus 1.39% 20 years ago), with similar incidence of intracranial or gastrointestinal events, and a trend for fewer fatal events. A significant difference is detected in the incidence of thrombotic complications that was as high as 3.5% annually in the 1997 report but fell drastically to 0.53% in the present report. The reduction is particularly evident in VTE patients, in whom thrombotic events fell from 4.8 to 0.8% annually (*p* = 0.01), and for events occurring during the first 3 months of treatment (*p* = 0.01) (Table 4). It is reasonable to surmise that the Italian anticoagulation clinics have markedly improved the way they manage patients, in particular those with recent VTE, by avoiding as far as possible periods of under-anticoagulation and improving the bridging with a parenteral anticoagulant especially during the first months of treatment. This improved management can in part be attributed to the education the Federation provides participant centers [31, 32]. An increased ability to perform more gradual and accurate induction phases of anticoagulation is a likely explanation for the much better results in the initial 3 months of VKA, a period that has been shown to be crucial for VKA-treated patients [33]. More patients died during treatment in the present (6.1%) versus previous report (3.7%) for causes apparently not correlated with the VKA treatment. This result is not surprising if we take into account the much higher prevalence of very elderly patients included in the present study.

In conclusion, the current ISCOAT 2016 study analyzing a large, inception-cohort of patients treated with VKA for different indications monitored in Italian anticoagulation clinics, reports very good clinical results, with rates of major bleeding and thrombotic complications lower than those reported in randomized trials and even in observational registries. When the current results are compared with those of the 1996 ISCOAT study, which had the same design and also involved anticoagulation clinics of the Italian FCSA Federation [1, 2], important differences are found: the age of treated patients is markedly increased in association with a drastic increase in patients treated for AF, while very few patients are treated for ischemic heart disease or other arterial diseases. Notwithstanding the older age of patients included in the current study, the rate of major bleedings and in particular of intracranial hemorrhages is similar in both studies. In contrast, a marked reduction in thrombotic complications is recorded, especially involving patients treated for VTE and during the first 3 months of treatment, pointing to a substantial improvement in the management of the initial phase of anticoagulation at our anticoagulation clinics, especially in VTE patients.
